# Development and standardization of multiplexed antibody microarrays for use in quantitative proteomics

**DOI:** 10.1186/1477-5956-2-9

**Published:** 2004-12-15

**Authors:** LT Perlee, J Christiansen, R Dondero, B Grimwade, S Lejnine, M Mullenix, W Shao, M Sorette, VT Tchernev, DD Patel, SF Kingsmore

**Affiliations:** 1 Molecular Staging Inc., 300 George St., New Haven, CT 06511 USA; 2 Thurston Arthritis Research Center and Department of Medicine, University of North Carolina, 3330 Thurston Building, Chapel Hill, NC 27599 USA; 3 National Center for Genome Resources, 2935 Rodeo Park Drive East, Santa Fe, NM 87505 USA

## Abstract

**Background:**

Quantitative proteomics is an emerging field that encompasses multiplexed measurement of many known proteins in groups of experimental samples in order to identify differences between groups. Antibody arrays are a novel technology that is increasingly being used for quantitative proteomics studies due to highly multiplexed content, scalability, matrix flexibility and economy of sample consumption. Key applications of antibody arrays in quantitative proteomics studies are identification of novel diagnostic assays, biomarker discovery in trials of new drugs, and validation of qualitative proteomics discoveries. These applications require performance benchmarking, standardization and specification.

**Results:**

Six dual-antibody, sandwich immunoassay arrays that measure 170 serum or plasma proteins were developed and experimental procedures refined in more than thirty quantitative proteomics studies. This report provides detailed information and specification for manufacture, qualification, assay automation, performance, assay validation and data processing for antibody arrays in large scale quantitative proteomics studies.

**Conclusion:**

The present report describes development of first generation standards for antibody arrays in quantitative proteomics. Specifically, it describes the requirements of a comprehensive validation program to identify and minimize antibody cross reaction under highly multiplexed conditions; provides the rationale for the application of standardized statistical approaches to manage the data output of highly replicated assays; defines design requirements for controls to normalize sample replicate measurements; emphasizes the importance of stringent quality control testing of reagents and antibody microarrays; recommends the use of real-time monitors to evaluate sensitivity, dynamic range and platform precision; and presents survey procedures to reveal the significance of biomarker findings.

## Background

Traditional immunoassay platforms have very limited multiplexing capability and high sample volume requirement. The development and application of high throughput, multiplex immunoassays that measure hundreds of known proteins in complex biological matrices, is becoming a significant tool for quantitative proteomics studies, diagnostic discovery and biomarker-assisted drug development [reviewed in [1-4]]. Two broad categories of antibody microarray experimental formats have been described: [1] direct labelling, single antibody experiments, and [2] dual antibody, sandwich immunoassays [4]. In the direct labelling method, all proteins in a complex mixture are tagged, providing a means for detecting bound proteins following incubation on an antibody microarray. In the sandwich immunoassay format, proteins captured on an antibody microarray are detected by a cocktail of detection antibodies, each antibody matched to one of the spotted antibodies. In addition, a variety of microarray substrates have been described, including nylon membranes, plastic microwells, planar glass slides, gel-based arrays and beads in suspension arrays. Much effort has been expended in optimizing antibody attachment to the microarray substrate. Finally, various signal generation and signal enhancement strategies have been employed in antibody arrays, including colorimetry, radioactivity, fluorescence, chemiluminescence, quantum dots and other nanoparticles, enzyme-linked assays, resonance light scattering, tyramide signal amplification and rolling circle amplification. Each of these formats and procedures has distinct advantages and disadvantages, relating broadly to sensitivity, specificity, dynamic range, multiplexing capability, precision, throughput, and ease of use [1-4]. In general, multiplexed microarray immunoassays are ambient analyte assays [5]. Given the heterogeneity of antibody array formats and procedures currently in use in proteomics studies, and the absence of a "gold standard", there exists an urgent need for development and adoption of standards that permit platform comparisons and benchmarking.

Unique, general considerations in assembling multiplexed immunoassays include: Requirements for elimination of assay cross-reactivity; configuration of multi-analyte sensitivities; achievement of dynamic ranges appropriate for biological relevance when performed in diverse matrices and biological states; and optimization of reagent manufacturing and chip production to achieve acceptable reproducibility. In contrast to traditional monoplex enzyme-linked immunoassays, generally agreed specifications and standards for antibody microarrays have not yet been formulated. A number of recent articles have started to examine certain of these issues [3,6,7].

**Figure 1:**
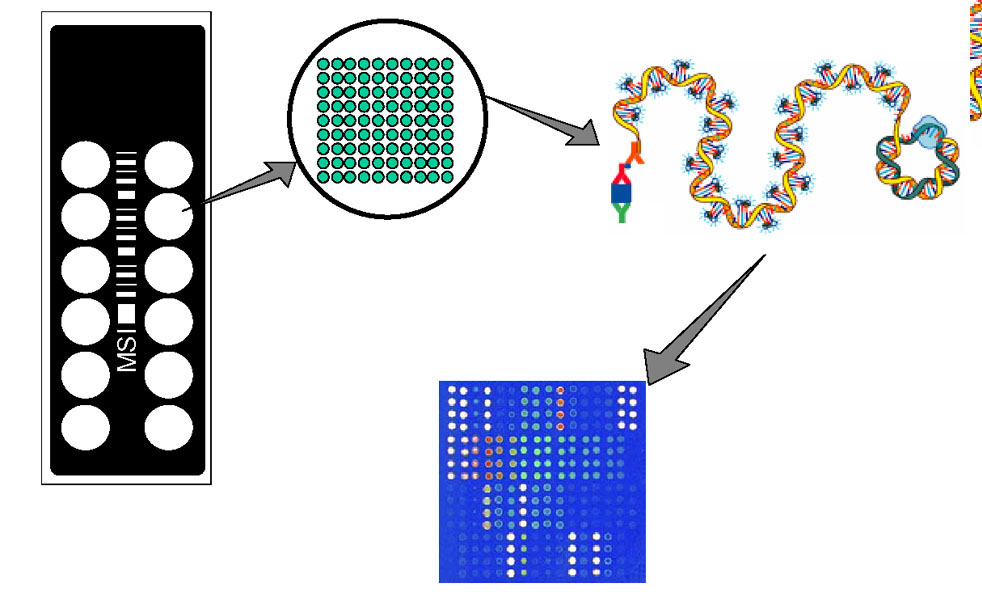
**Schematic layout of antibody microarray slide and RCA immunoassay. **At the far left is an illustration of the 1" × 3" slide platform containing sixteen individual sample wells with an etched barcode. Within each of the wells, a 16 × 16 configuration of printed capture antibodies is arrayed. Each of the capture antibodies is capable of binding analytes from applied samples and undergoing RCA signal amplification. Finally, the fluorescently labeled signal, detected through conventional laser scanning, is quantified.

Microarray immunoassays performed on planar glass slides and employing signal enhancement with rolling circle amplification (RCA), have been developed by several groups and have demonstrated usefulness in measurements of temporal and dose-dependent changes in a variety of immunological model systems and human diseases [[1,2,8-16]; Patel, D.D. et al. Submitted]. In general, these RCA microarray immunoassays have utilized indirect sandwich immunoassays featuring five steps (Figure 1):

I. Analytes in an applied sample bind to capture antibodies immobilized on a silanized glass surface.

II. Applied secondary biotinylated detector antibodies bind to captured analytes, creating a highly specific immune complex.

III. Biotinylated detector antibodies bound to the immune complex are detected with a universal anti-biotin antibody. The latter is conjugated to primer oligonucleotides that are pre-annealed to a complementary circular oligonucleotide.

IV. DNA polymerase extends the 3' ends of primers around the circles, resulting in long, single stranded RCA products that remain attached to the complex.

The RCA product, composed of tandem DNA repeats complementary to the circle sequence, is detected by hybridization with cyanine 5 (Cy5)-labeled complementary oligonucleotides.

The present report describes initial development of standardized operating procedures, quality controls and standards for microarray immunoassays performed on planar glass slides using signal enhancement with RCA. These metrics have been tested for use in generation of data with adequate sensitivity, reproducibility and assay performance for biomarker discovery [[12-14,16], Patel D.D et al., submitted]. Initial specifications and standards are also described for the addition of new analytes to antibody microarrays, which are needed to ensure that a high level of performance is maintained. While certain of these recommendations and standards are specific to RCA immunoassays, others represent generally applicable first generation standards for benchmarking antibody array platforms that enable interoperability of data generated in proteomics studies.

## Results

### Data Quality

To demonstrate the feasibility of using a multiplex immunoassay system to measure protein levels in complex biological matrices, the performance of dual-antibody, sandwich immunoassay arrays performed on planar glass slides with RCA signal enhancement was evaluated for specificity, sensitivity, reproducibility and accuracy using standardized titrations, spiked biological matrices and clinical samples. Array performance was evaluated based on ability to: measure analytes across a broad dynamic range at sufficiently low coefficients of variation (CVs); detect proteins at levels requisite to capture biologically relevant expression differences; confirm reliability of methods to normalize data to minimize platform imprecision and demonstrate the utility of generating standard curves to convert analyte MFI (mean fluorescence intensity) data into mass unit information.

#### Data Redaction

An advantage of arrays is the ability to measure each analyte multiple times, enhancing precision. Capture antibody spots were printed in quadruplicate on planar glass slides providing redundancy of individual analyte measurements. Data redaction was applied to raw immunoassay data to improve data quality by eliminating outlier data points. Outliers were identified by employing two subsequent statistical approaches in a step-wise manner.

**Figure 2:**
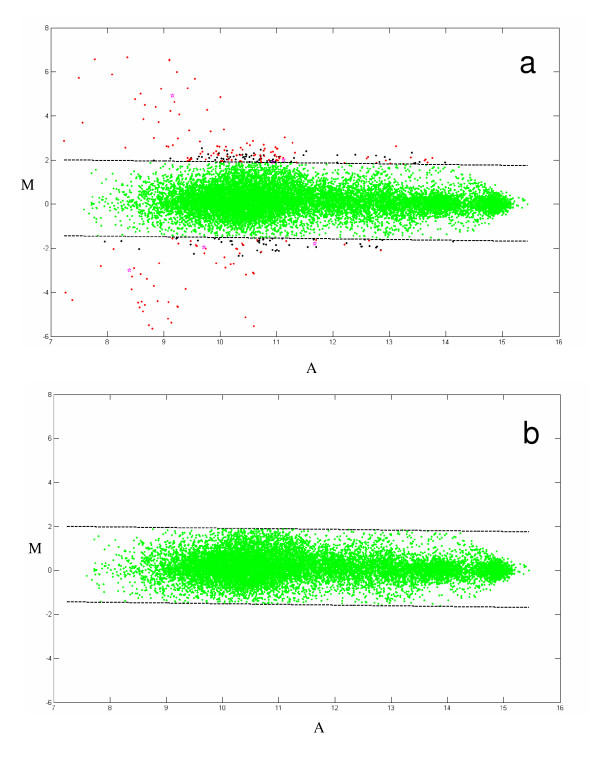
**An example of raw data quality and outlier removal. ***(panel a, top) *Raw data (37 analytes) from array 4 containing all sample replicates shown on an MvA plot (a typical microarray data plot of the log ratio vs. the log difference for each pair of intensities. See: Dudoit, S., Yang, Y. H, Callow, M. J., and Speed, T. P. (2002) *Statistica Sinica ***12**, 111–140). The dashed lines indicate a 99% confidence interval around the data and outliers of this interval are shown in red, black or magenta. *(panel b, bottom) *Redacted data with 1% of outlier data removed (all points outside of the displayed confidence interval).

First, the Bland-Altman plot was used. Bland-Altman plots are often used in DNA microarray analysis to identify differences and/or replicate outliers. This involves plotting the difference between the logarithm of intensities of two replicates (M) versus the average of logarithm of intensities (A) for each analyte within an individual array (see Material and Methods). Thus, there will be 6 MvA plots for each data set to reflect the 170 analytes positioned across 6 arrays. Each MvA plot will contain 3*Ns*Na points, where 3 reflects the number of possible unique pair wise combinations of the three replicates, Ns represents the number of samples and Na defines the number of analytes measured on a given array. An example of an MvA plot produced in a project comprising 150 clinical serum samples for Array 4 with 37 analytes is shown in Figure 2 (panel a). This plot contains 3*150*37 = 16650 data points. The quadruplicate measurements within an arrayfor each anlayte are represented as a mean replicate value.. Lines represent 99% confidence intervals for individual data points. Data points outside of 99% confidence intervals are considered outliers. The quality and /or intensity of individual spots are manually investigated for each outlier by using proprietary visualization software, which allows examination of individual spot image/quality at every data processing step. Outliers are redacted by removing aberrant spots from the data set. The resulted MvA plot is shown on Figure 2 (panel b).

**Figure 3:**
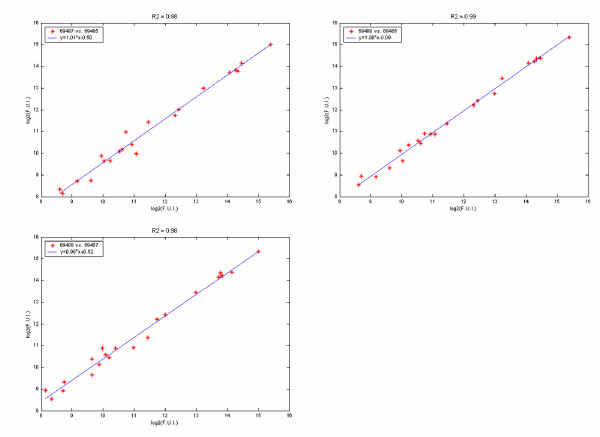
Pair wise scatter plots between three replicates of a sample. Each replicate was assayed on a different slide. Solid lines represent linear regression fits. Regression equation is indicated within legend box along with the individual slide barcodes for this particular assay. R^2 ^value of the fit is indicated in the title. Both X and Y-axes indicate mean fluorescence signal Log_2_(MFI).

The second step of data reduction involves a linear correlation analysis. Pair-wise correlation analysis is done between all replicates of individual sample. Figure 3 shows the three scatter plots generated for the three replicates of a representative sample. The correlation coefficient (R^2^) is examined for each plot. Each plot contains Na data points, where Na reflects the number of analytes. Plots with R^2 ^values <0.95 are examined to identify the cause of the poor correlation. We have identified two major sources of poor correlation: incorrect positioning of the capture grid during image quantitation and general aberrations in image spot quality. Assigning the specific source of low correlation is accomplished by tracing back to the image data. In the case of grid misplacement, suspect data images are re-quantified. Poor correlations due to aberrant spot morphology/intensity are manually examined and removed from data set. If the R^2 ^value does not improve as a result of outlier removal, the replicate is redacted from the data set. The sample is considered passed if there are two replicates with R^2 ^>= 0.95. The pass rate is defined as the number of passed samples divided by the total number of samples. A run of an array is considered to be passed if 85% of the samples have two or more passed replicates.

In our experience, applying the MvA statistical approach first, followed by the linear correlation analysis is an efficient process to identify outliers without compromising data throughput. Since, MvA plots can be generated quickly, it allows for relatively fast redaction of significant outliers using an objective semi-automated approach. In contrast, the sample correlation analysis is considerably more labor intensive and currently requires manual investigation of each scatter plot with R^2 ^values < 0.95. This process reduces throughput of data redaction, particularly on large data sets. Table 1 shows the impact of using MvA plot analysis as a first step approach for outlier removal in a clinical project containing 106 samples. Each assay in Table 1 represents a single sample replicate, with a total possible number of assay points equal to 418 (106*3 = 418). The table reflects an assay count of 417 due to one sample having only two replicates due to a shortfall in sample volume. The correlation analysis performed on all sample replicates increased the pass rate by 8% following outlier removal. This improvement was due to the elimination of individual analyte replicates having a negative impact on total sample correlation derived from all analyte replicates within an array (Table 1).

**Table 1:** Improved sample pass rates achieved through individual analyte data reduction

	**Before**	**After**	**Total**
**Assays (#)**	357	393	417
**Assays (%)**	86	94	100

In general, for data sets with more than 40 samples, outlier removal only demonstrated small improvements in reducing average CVs across all samples. The most significant impact of outlier removal is on improving reproducibility across the three replicates of the individual samples. In our experience, outlier removal has been shown to reduce individual sample replicate CVs by 2–3 fold. This effect is directly related to improving sample correlation pass rates by by 10–20%.

#### Normalization

Many systematic factors can modify spot intensity during the process of measurement. Normalization is the process of reducing the effects of systematic variation on spot intensity. Normalization in DNA microarrays typically involves adjusting distributional summaries of data (mean, median) from each chip to common reference values. For example, one assumption could be that the average signal from each protein chip should be the same, as with DNA microarrays and the difference between replicate values is due to systematic variability in the measurement process. Unfortunately, the nature of protein antibody microarrays, configured with a multiplex of individual capture and detector antibodies, is more specialized and differentiated than that of a DNA microarray. Use of a single reference factor derived from a global value is not sufficiently refined to take into account the difference in platform configuration. In the current report, the organization of protein microarrays allows the measurement of up to 16 samples per slide (chip). This is very different from DNA microarrays where one chip represents the total collection of measured values for an individual sample.

To accommodate the differences inherent to the platform, we have applied a normalization strategy based on the three major sources of technical variability observed in our system. The first type of variability relates to spot-to-spot differences observed between quadruplicate spots of the individual analytes printed within a sample well. The second level of variability can be described as the difference in measurements between wells within the same slide. The third element of variability represents the variability observed between sample wells compared across different slides. We found that slide-to-slide variability is the largest source of variation accounting for more than 70% of the total measurement imprecision (data not shown). Thus the goal of normalization is to reduce the imprecision of slide-to-slide measurement error since this represents the major source of platform variability.

Normalization is performed using a system of standard controls to reduce the effect of slide-to-slide variability. A series of four standard control samples (see "Anchor Point Calibrators" in Methods and Materials) are run in 4 wells of each slide. Each control sample represents a cocktail of the full repertoire of analytes for the given array tittered at 4 specific concentrations. The standards have been optimized at concentrations (12 pg/ml, 111 pg/ml, 333 pg/ml and 1000 pg/ml) to capture measurements across the linear range of detection for each analyte. The global average of total analyte signal for the four prepared controls is calculated across all slides run in a batch. An adjustment factor is created for each slide that reflects the difference between global intensity average for all slides and the individual intensity average based on the controls from the individual slide. The averaged pixel intensity of each spot on the slide is scaled by the adjustment factor.

**Figure 4:**
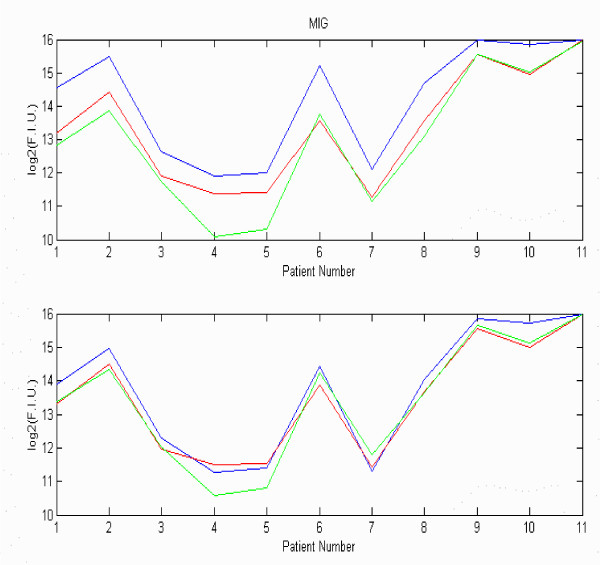
**Effect of Normalization. **The top panel reveals the raw data, shown as the Log_2_(MFI), for the analyte Monokine induced by interferon gamma (MIG) across three replicates with 11 patient samples (numbered on the X-axis). The bottom panel reflects the impact of normalization in reducing variation in intensity within each sample and hence the replicate MFI CV.

As an example, the average value of the adjustment factor was evaluated across a batch of 33 slides and found to have a value of 1.33+/- 0.47. The primary benefit of normalization was in reducing the replicate sample CVs. Figure 4 contains two panels revealing the impact of normalization on individual analyte CVs across a series of samples for a given analyte. The upper panel shows the variation in raw MFI signal intensities on a logarithm scale observed between the 3 replicate measurements for each of the 11 samples. The lower panel reveals the impact of normalization on reducing variability. Normalization typically reduced sample replicate CVs an average of 5% without producing rank order changes in analyte MFI.

#### Assessment of Platform Precision

A 15-point series of standardized titrations containing recombinant proteins diluted in buffer were used to evaluate platform precision. This assessment was used in the quality control of each slide lot prior to release, as well as within each client project to verify run-time analyte performance. Six replicates for each point were run in the quality control testing of each slide lot and six replicates of each point were run within each client study to generate standard curves. CVs were evaluated for each concentration of analyte across six slides. Average CVs were calculated for each analyte. Statistical summaries of CV distribution across all array 2 analytes using the standardized 15-point standard titration series are shown in Table 2. The mean CVs of the control titration replicates were typically in the 10–15% range following normalization. Collectively evaluating mean, median and interquantile range CVs served to identify measurements significantly influenced by outlier values producing a skewed distribution. In general, CVs obtained for the quadruplicate within-well analyte measurements were 5–9% for the prepared controls. Replicate sample CVs obtained from biological samples tended to be somewhat higher than prepared controls with quadruplicate within well measurements at 10–15% post normalization and 20–25% average CVs for replicates samples positioned in wells across different slides. Table 3 reveals CVs obtained in a project containing 110 clinical serum samples run across the 6 arrays. Each sample was tested in triplicate generating 3 replicates measurements obtained from 3 different slides. The average CVs were 18%, 20%, 17%, 20%, 16% and 17 % for Arrays 1, 2, 3, 4, 5 and 6 respectively. The data reduction rate was less than 5% of all data points. This reduction rate is typical of what we have observed across more than 30 clinical projects.

**Table 2:** Mean and standard deviation of analyte MFI CVs from titration standards for a given array

	**%CV**				
**Conc. (pg/ml)**	N*	**Maximum**	**Mean**	**Minimum**	**Std. Dev.**

**12**	26	19.4	14.0	9.4	2.4
**111**	26	15.8	9.4	6.1	2.9
**333**	26	15.3	9.4	4.5	2.5
**1,000**	26	29.1	12.0	2.6	5.8
**3,000**	26	26.4	9.7	2.5	6.2
**9,000**	26	22.2	7.8	2.8	4.3
**27,000**	26	16.3	8.2	2.5	4.0
**81,000**	26	19.7	7.8	2.6	4.1

* N reflects 26 analytes measured on array 2.

**Table 3:** Mean and standard deviation of MFI CVs from clinical samples

**Array**	N*	**<CV>**	**Std Dev**
**1**	3856	18.1	11.2
**2**	3752	19.7	12.3
**3**	3891	16.9	10.6
**4**	5211	20.4	15.4
**5**	3750	16.2	11.2
**6**	4274	17.4	10.9

* N reflects all analyte measurements for all QC passed sample replicates (project contained 110 samples tested in triplicate).

#### Variance Decomposition

**Figure 5:**
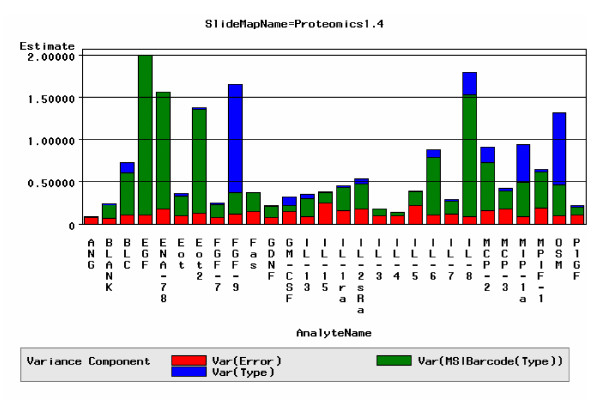
**Variance Decomposition**. Example of a variance decomposition analysis performed on the analytes for array 1 in a client study. The X-axis corresponds to analyte name and y-axis corresponds variance. Red blocks reflect platform variation, while green and blue blocks represent inter-patient and inter-treatment variance respectively.

A variance decomposition analysis was performed to reveal the extent to which platform error influenced the ability to identify biomarkers. The variance component assigned to platform error was typically found to be an order of magnitude lower than the average inter-individual variation. Figure 5 reveals the contribution of platform error on the total variance observed in a given project for each analyte across array 1. These results indicated the system variability was sufficiently low to capture moderate expression level differences that were reflective of biological change.

#### Average lower limit of quantitation (LLQ)/ upper limit of quantitation (ULQ) values and analyte dynamic range

**Figure 6:**
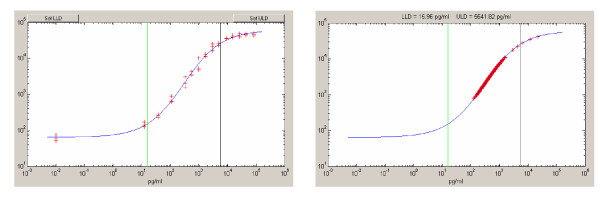
**LLQ/ULQ**. (*Left*) Plot of 3 replicate points from a 15-point titration series of IL-8. The LLQ is indicated by the green vertical line and the ULQ indicated by the rightmost black vertical line. The zero point was removed from the curve fitting procedure since the data undergoes a log transformation. The right panel reveals sample values that fell within and above dynamic range of assay. Here, the majority of tested points for IL-8 fell within the LLQ/ULQ dynamic range.

The left panel of Figure 6 shows a typical dose response curve of MFI (mean fluorescence intensity) versus analyte concentration, generated for a single analyte based on the 3 replicate measurements from the 15-point titration series containing a multiplex of recombinant analytes spiked into buffer at fixed concentrations. Each titration point was replicated across 3 control slides generating 3 replicate measurements. The vertical lines defined the LLQ and ULQ as well as the dynamic range of the individual analyte within a 30% CV of analyte concentration. The right panel of Figure 6 shows the corresponding clinical sample values obtained in the same run revealing the sample values that fell within, above and below the linear range of detection as defined by the standard titrations. Table 4 contains a summary of the average dynamic range obtained for the 170 analytes surveyed over 8 independent runs.

**Table 4:** Average dynamic range achieved across each of the production arrays

	**Detectable^1 ^((W+A)>50%)**	**> 1 log**	**> 1.5 log**	**> 2 log**	**> 2.5 log**	**> 3 log**
**Array 1**	59%	96%	78%	41%	0%	0%
**Array 2**	65%	100%	92%	77%	12%	0%
**Array 3**	67%	100%	70%	33%	7%	0%
**Array 4**	92%	97%	92%	54%	16%	3%
**Array 5**	100%	92%	80%	48%	20%	0%
**Array 6**	85%	100%	74%	41%	7%	0%
***Average***	78%	98%	81%	49%	10%	1%

Data shown is based on a summary of 8 independent project runs. 98% of the analytes demonstrated at least 1 log dynamic range, 49% of the analytes demonstrated at least a 2 log dynamic range, 10% at least a 2.5 log dynamic range and 1% with at least a 3 log dynamic range.

^1 ^See legend table 5–10 for explanation.

#### Performance assessment

A performance assessment of individual analytes was conducted to determine the utility of each analyte across multiple projects covering diverse disease areas. Each analyte was evaluated according to the percentage of clinical samples that fell within (W), below (B) or above (A) the linear range of detection. (Tables 5,6,7,8,9,10) Analytes were considered to be detectable if the percentage of samples that fell in the W+A categories was greater than 50%. The projects surveyed across 8 independent studies containing over 1,000 clinical samples. The disease areas included rheumatoid arthritis, osteoarthritis, systemic lupus erythematosus (SLE), chronic obstructive pulmonary disease (COPD), asthma, diabetes and ovarian cancer. The average percentage of detectable analytes was 56% for array 1, 62% for array 2, 67% for array 3, 73% for array 4, 81% for array 5 and 85% for array 6 across the 8-project survey group. A limited number of analytes (<5%) revealed high endogenous concentrations, producing assay saturation where >90% of the measured samples fell above the linear range of detection. In most cases, this could be resolved by re-running a sample dilution or scanning at a lower gain. Although there were analytes that had detectable percentages below 50%, in many cases these reflected analytes that were only detected under up-regulated conditions associated with specific disease states or conditions of drug induction, revealing value within specific disease or therapeutic areas. Tables 5,6,7,8,9,10 also reveal the average LLQ/ULQ values of the 170 analytes within a 30% CV of concentration obtained from the control titrations run in parallel with the clinical samples.

**Table 5:** Array 1: Averaged LLQs/ULQs in pg/mL obtained from 15 point standard titrations

**Feature**	**B**	**W**	**A**	**W+A**	**LLQ (pg/mL)**	**ULQ (pg/mL)**
**ANG**	1	6	93	99	7	1262
**BLC**	7	93	0	93	62	4614
**EGF**	32	65	3	68	347	1184
**ENA-78**	8	81	12	92	161	9225
**Eot**	19	81	0	81	186	4454
**Eot2**	2	83	15	98	23	2313
**FGF-7**	28	72	0	72	218	19367
**FGF-9**	67	33	0	33	463	21049
**Fas**	16	84	0	84	290	39248
**GDNF**	66	34	0	34	48	7715
**GM-CSF**	67	33	0	33	67	3626
**IL-13**	29	71	0	71	29	4458
**IL-15**	73	27	0	27	4682	52224
**IL-1ra**	45	54	0	55	71	8960
**IL-2sRa**	3	94	3	97	20	3975
**IL-3**	58	42	0	42	852	19428
**IL-4**	81	19	0	19	43	3548
**IL-5**	67	33	0	33	13	2438
**IL-6**	59	34	7	41	14	2291
**IL-7**	73	27	0	27	32	2396
**IL-8**	21	65	13	79	6	916
**MCP-2**	13	84	4	87	42	2144
**MCP-3**	45	54	1	55	58	3205
**MIP-1a**	51	46	3	49	464	10315
**MPIF-1**	9	91	0	91	293	7410
**OSM**	73	27	0	27	78	8511
**PlGF**	46	54	0	54	61	3080

Data shown is from titrations run in 8 client projects (1000 clinical samples surveyed) covering 8 diverse disease areas. The percentage of samples falling below (B), within (W) or above (A) the linear range of detection are presented. The analyte was considered to be detectable if the W+A percentage was above 50%.

**Table 6:** Array 2: Averaged LLQs/ULQs in pg/mL obtained from 15 point standard titrations

**Feature**	**B**	**W**	**A**	**W+A**	**LLQ (pg/mL)**	**ULQ (pg/mL)**
**AR**	73	27	0	27	48	5599
**BDNF**	8	87	5	92	26	3956
**Flt3Lig**	2	98	0	98	17	9835
**GCP-2**	26	74	0	74	132	13153
**HCC4**	8	46	46	92	112	7907
**I-309**	30	70	0	70	20	5142
**IL-17**	95	5	0	5	330	7921
**IL-1a**	69	31	0	31	10	2943
**IL-1b**	46	54	0	54	4	2932
**IL-2**	89	7	4	11	31	3671
**M-CSF**	55	45	0	45	86	6761
**MCP-1**	17	69	14	83	82	2065
**MIG**	8	87	5	92	13	5030
**MIP-1b**	29	56	16	71	16	2399
**MIP-1d**	10	72	18	90	192	6969
**NT-3**	75	25	0	25	160	22314
**NT-4**	60	40	0	40	128	19170
**PARC**	4	25	70	96	12	1870
**Rantes**	0	13	87	100	5	1302
**SCF**	29	71	0	71	69	20306
**TARC**	15	85	0	85	21	3134
**TNF-R1**	2	97	1	98	108	16285
**TNF-a**	71	29	0	29	56	6901
**TNF-b**	88	12	1	12	221	7617
**VEGF**	86	14	0	14	702	79564
**sgp130**	0	63	37	100	334	38573

Data shown is from titrations run in 8 client projects (1000 clinical samples surveyed) covering 8 diverse disease areas. The percentage of samples falling below (B), within (W) or above (A) the linear range of detection are presented. The analyte was considered to be detectable if the W+A percentage was above 50%.

**Table 7:** Array 3: Averaged LLQs/ULQs in pg/mL obtained from 15 point standard titrations

**Feature**	**B**	**W**	**A**	**W+A**	**LLQ (pg/mL)**	**ULQ (pg/mL)**
**BTC**	84	16	0	16	980	25361
**DR6**	7	93	0	93	1200	86864
**FGF1**	60	40	0	40	162	53854
**FasL**	79	21	0	21	4300	82436
**Fractalkine**	78	22	0	22	662	12170
**GROb**	6	91	3	94	66	2505
**HCC1**	3	28	70	97	164	3610
**HGF**	70	30	0	30	1039	80079
**HVEM**	6	94	0	94	636	105560
**ICAM-3**	0	100	0	100	477	126635
**IGFBP2**	0	40	60	100	1523	46325
**IL2Rg**	71	29	0	29	429	41325
**IL5Ra**	63	37	0	37	1945	57281
**IL-9**	36	64	0	64	4506	156041
**L-Selectin**	0	47	53	100	161	28007
**Leptin**	5	69	26	95	1322	58408
**MCP4**	21	74	6	79	93	2508
**MIP3b**	18	82	0	82	40	9211
**MMP7**	0	99	1	100	70	21138
**MMP9**	0	51	49	100	3043	163481
**PECAM1**	6	93	1	94	1033	52935
**RANK**	17	83	0	83	223	87693
**SCF R**	3	97	0	97	445	38465
**ST2**	51	49	0	49	558	66234
**TIMP1**	0	77	23	100	2010	90647
**TRAIL R4**	87	13	0	13	4945	122116
**VEGF R2**	17	83	0	83	1254	138750

Data shown is from titrations run in 8 client projects (1000 clinical samples surveyed) covering 8 diverse disease areas. The percentage of samples falling below (B), within (W) or above (A) the linear range of detection are presented. The analyte was considered to be detectable if the W+A percentage was above 50%.

**Table 8:** Array 4: Averaged LLQs/ULQs in pg/mL obtained from 15 point standard titrations

**Feature**	**B**	**W**	**A**	**W+A**	**LLQ (pg/mL)**	**ULQ (pg/mL)**
**ALCAM**	0	100	0	100	991	145528
**CD27**	10	90	0	90	508	148468
**CD30**	53	47	0	47	2460	128280
**CTACK**	0	100	0	100	43	10691
**Eot-3**	37	63	0	63	130	28149
**FGF-2**	31	67	2	69	102	5814
**FGF-4**	57	43	0	43	260	14563
**Follistatin**	8	92	0	92	138	57120
**GRO-g**	12	75	13	88	59	4344
**I-TAC**	11	89	0	89	16	6191
**ICAM-1**	1	76	23	99	289	29018
**IFN-g**	39	60	1	61	14	7365
**IFN-w**	37	58	5	63	1177	42508
**IGF-II**	0	85	15	100	46	13538
**IGF-1R**	33	67	0	67	330	91601
**IGFBP-1**	2	72	26	98	272	85578
**IGFBP-3**	0	7	93	100	5530	30760
**IGFBP-4**	0	80	20	100	410	22880
**IL-1 sR1**	30	70	0	70	534	54857
**IL-10rb**	12	88	0	88	28	11331
**IL-16**	28	72	0	72	724	86874
**IL-1 srII**	19	81	0	81	509	76440
**IL-2rb**	71	29	0	29	10277	107828
**LT bR**	14	86	0	86	34	37957
**Lymphotactin**	28	72	0	72	166	9216
**M-CSF R**	0	91	9	100	1951	121318
**MIP-3a**	16	84	0	84	13	3389
**MMP-10**	10	90	0	90	158	42033
**PDGF-Ra**	30	66	5	70	8112	151722
**PF4**	0	23	77	100	67	5458
**TGF-a**	33	67	0	67	35	2678
**TIMP-2**	0	12	88	100	130	25224
**TRAIL R1**	33	67	0	67	21	10956
**VAP-1**	1	23	76	99	4624	150226
**VE-cadherin**	1	94	5	99	2652	146826
**VEGF-D**	34	66	0	66	1370	100539
**b-NGF**	27	73	0	73	141	12400

Data shown is from titrations run in 8 client projects (1000 clinical samples surveyed) covering 8 diverse disease areas. The percentage of samples falling below (B), within (W) or above (A) the linear range of detection are presented. The analyte was considered to be detectable if the W+A percentage was above 50%.

**Table 9:** Array 5: Averaged LLQs/ULQs in pg/mL obtained from 15 point standard titrations

**Feature**	**B**	**W**	**A**	**W+A**	**LLQ (pg/mL)**	**ULQ (pg/mL)**
**4-1BB**	46	54	0	54	334	92233
**ACE-2**	37	63	0	63	1138	128330
**AFP**	4	96	0	96	17	11483
**AgRP**	18	82	0	82	72	13198
**CD141**	0	62	38	100	780	14852
**CD40**	30	70	0	70	101	19145
**CNTF Ra**	18	82	0	82	46	21212
**CRP**	4	28	68	96	201	12376
**D-Dimer DD5**	0	4	96	100	12070	65452
**E-Selectin**	0	85	15	100	89	21531
**HCG**	40	59	0	60	345	18736
**IGFBP-6**	0	2	98	100	855	38589
**IL-12p40**	44	56	0	56	2505	159213
**IL-18**	0	100	0	100	5	3743
**LIF Ra**	45	54	1	55	5350	117637
**MIF**	3	84	13	97	9753	132966
**MMP-8**	0	82	18	100	111	48374
**NAP-2**	0	19	81	100	103	9114
**Neut Elast**	0	66	34	100	376	24009
**P-Selectin**	0	82	18	100	2128	93967
**PAI-II**	28	72	0	72	251	115374
**Prolactin**	0	96	4	100	1133	77120
**Protein C**	0	83	17	100	1266	154054
**Protein S**	0	1	99	100	10808	64646
**TSH**	43	57	0	57	81	15614

Data shown is from titrations run in 8 client projects (1000 clinical samples surveyed) covering 8 diverse disease areas. The percentage of samples falling below (B), within (W) or above (A) the linear range of detection are presented. The analyte was considered to be detectable if the W+A percentage was above 50%.

**Table 10:** Array 6: Averaged LLQs/ULQs in pg/mL obtained from 15 point standard titrations

**Feature**	**B**	**W**	**A**	**W+A**	**LLQ (pg/mL)**	**ULQ (pg/mL)**
**6Ckine**	0	100	0	100	152	27541
**ACE**	0	60	40	100	3283	93777
**CA125**	19	81	1	81	273	120834
**CNTF**	67	33	0	33	2562	109254
**ET-3**	9	83	8	91	881	25735
**Endostatin**	0	17	83	100	432	9966
**ErbB1**	1	95	4	99	3864	86553
**ErbB2**	12	88	0	88	2293	118586
**FGF R3 (IIIb)**	47	53	0	53	455	89028
**FGF R3 (IIIc)**	53	47	0	47	214	51823
**FGF-6**	42	58	0	58	220	18535
**G-CSF**	69	31	0	31	1487	49342
**HB-EGF**	41	59	0	59	47	3143
**IFN-a**	41	59	0	59	20	5021
**LIF**	52	48	0	48	655	52660
**MMP-1**	3	97	0	97	537	103951
**MMP-2**	0	99	1	100	1446	154542
**OPN**	0	97	3	100	496	60615
**PAI-1**	0	10	90	100	22	8289
**PDGF Rb**	14	86	0	86	645	59309
**PEDF**	0	19	81	100	2599	63199
**TGF-b RIII**	10	50	40	90	595	13518
**Tie-2**	43	57	0	57	6005	147662
**VEGF R3**	10	90	0	90	466	37150
**uPA**	12	88	0	88	89	16374
**uPAR**	11	89	0	89	1259	125972
**VCAM-1**	0	49	51	100	1577	150401

Data shown is from titrations run in 8 client projects (1000 clinical samples surveyed) covering 8 diverse disease areas. The percentage of samples falling below (B), within (W) or above (A) the linear range of detection are presented. The analyte was considered to be detectable if the W+A percentage was above 50%.

### Validation of Array Performance

The development of an antibody array featuring 25–40 novel immunoassays requires extensive validation related to the comprehensive assessment of antibody cross reactivity, definition of analyte minimal detection limits (MDL) and establishing robust assay performance. Each antibody array must be validated for use with several matrices, since the latter may have different ambient analyte levels (and therefore, different ideal MDL) or cross-reactivity profiles.

#### Analyte sensitivity

Analyte sensitivity was assessed to identify analytes lacking adequate performance for retention on an array. Additional experiments were performed to determine the endogenous levels of each analyte. For analytes without previously reported biological values, the "0 × n" assays indicated the approximate ambient analyte level. Testing across multiple biological matrices was required, since different matrices affected the detection of analyte specific signals. The "0 × n" experiments also revealed the level of non-specific background which was influenced by the total concentration of antibody load in the detector mix. In our experience, certain plasma matrices were also more likely to generate high background when compared to matched serum samples. The impact of high generalized background is a reduced sample pass rate. When background was observed, the total detector antibody concentration could often be reduced to minimize background noise. Ultimately, a balance between reduction in background and enhancement of sensitivity was required to achieve maximal analyte performance in a mutiplex configuration.

#### Analyte cross reaction

The results of the 1 × (n-1) assays identified analytes that demonstrated cross-reaction between the captured analyte and the complex detector mix prepared without the cognate detector antibody. Binding between the spiked analyte and the cognate capture that generated signal, indicated a cross-reaction to one or more non-cognate detector antibodies contained within the complex mix. In cases where non-cognate detector signal was observed, an additional series of experiments were run with the corresponding analyte tested against each of the individual detectors to identify the cross-reacting detector antibody. Since cross reaction is an additive process, the outcome of the cross reaction assessment allowed for adjustments to be made to achieve a balance between maximizing content with multiplexed array specificity. The 0 × (n-1) assays were run to provide a baseline of MFI values to compare to the results obtained in the 1 × (n-1). In addition, the 0 × (n-1) experiments also served to screen the various biological matrices for cross-reactivity with endogenous proteins.

#### Analyte performance under multiplexed conditions

**Figure 7:**
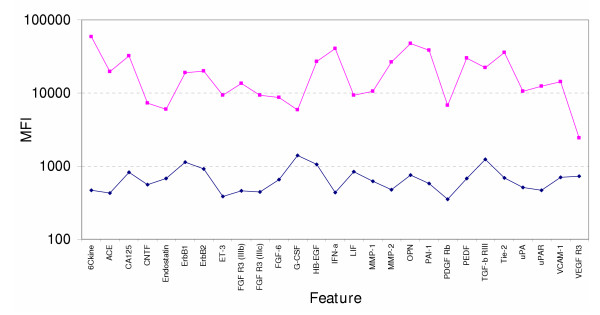
**New array validation**. Stress testing at 50X MDL analyte concentration. The pink line reveals the specific MFI signal for each analyte at 50X MDL in the presence of all detectors (n × n). The blue line shows the signal for each analyte under conditions where all analytes are added at 50X MDL along with all detector antibodies minus the cognate detector antibody (n × (n-1)) to reveal non-specific signal contributed by non-cognate detectors.

Serum MDLs were typically found to be higher than buffer MDLs due to the presence of endogenous analyte, potential analyte-binding proteins present in the biological matrix and other possible matrix-related interferences. The assay conditions used to stress test the system under conditions of high analyte load were designed to identify cross-reaction thresholds for each of the individual analytes. MFI cut off values were used to identify significant increases in non-cognate signal that warranted removal of a feature from the array. The results provide a certain utility in predicting array performance under conditions where sample analyte concentrations exceed reported biological levels. Examples might include patient samples tested under diseased states, elevated analytes produced in stimulated cell culture supernatants or in samples exhibiting a strong drug response. The final validation involved measuring the accuracy of the multiplex assay when challenged with a high concentration of analyte. Figure 7 shows the correlations of signal intensities obtained between (1 × n) compared to (n × n) experiments at 50x MDL levels. High R^2 ^values obtained between the two conditions provided a measurement of the accuracy of the multiplexed system.

## Discussion

Thirty years of widespread use of conventional, monoplex immunoassays has established firm benchmarks for performance in protein measurement. In the present paper, we have examined several, unique but general considerations in assembling multiplexed immunoassays with performance similar to conventional monoplex immunoassays. These include development of a comprehensive validation program to identify and minimize antibody cross reaction under highly multiplexed conditions; application of standardized statistical approaches for data handling for highly replicated assays; inclusion of standardized samples in each run to normalize sample replicate measurements; quality control of reagents and antibody microarrays; implementation of real-time monitors to evaluate sensitivity, dynamic range and platform precision; and initial procedures for identification of specific, significant immunoassay results in biomarker discovery projects involving clinical samples. Each of these will be discussed briefly.

### Requirement for a comprehensive array validation program

An array validation program represents the foundation of tests required to establish robust assay performance in a multiplexed environment. The most significant component in array validation is the comprehensive evaluation of cross reactivity. The vast majority of the ~5000 commonly available antibody pairs available today have not previously been evaluated for cross reactivity in a multiplexed environment. Therefore, the recommended program should include procedures that identify analytes demonstrating cross reactivity with immobilized capture antibodies as well as cross reaction that might manifest between the secondary detector antibody with a non-cognate analyte or non-specific binding to an immobilized capture agent.

The performance of analytes in a multiplexed configuration should be benchmarked against the baseline, monoplex performance. This multiplexed immunoassay comparison with baseline performance, together with minimal standards for multiplexed cross-reactivity, permits determination of the practical, optimal number of array elements that can be successfully combined. In our experience, using dual-antibody, sandwich immunoassays, planar glass slides and RCA signal amplification, protein micorarrays can generally accommodate multiplexing of 25–35 analytes without an appreciable drop in individual analyte sensitivity or performance. Specifically, we have described development of six different dual-antibody sandwich immunoassay arrays, each containing 25–37 sandwich immunoassays. Since cross reactivity is an additive process, the ultimate goal is to achieve a balance between maximal multiplexing and monoplex-like performance. With exhaustive selection for antibodies without cross-reactivity in multiplexed format, it is possible to multiplex 50 sandwich immunoassays. However, this exercise is very expensive. In our experience, suspension arrays, alternative microarray surface substrates and attachment chemistries do not offer significant advantages in multiplexing while maintaining performance. We have not evaluated the impact of novel, affinity ligands on multiplexing.

Additional array validation for cross-reactivity should include "stress-testing" under high analyte load to reflect conditions where analytes may be significantly over-expressed. In our experience, levels of induction of proteins in common biological matrices can be very large following drug administration or in disease states, and may induce cross-reactivity that is not observed in testing within normal biological analyte levels.

Finally, array validation should be performed across all common sample matrices to examine effects on assay performance associated with endogenous analyte, matrix specific analyte binding proteins or other matrix-specific inhibitors. Absence of cross-reactivity for an immunoassay in one matrix does not always imply absence of cross-reactivity in others. The matrices for which the antibody microarrays described herein have been validated include isotonic buffers, serum, citrate plasma, heparin plasma, EDTA plasma, cell culture supernatants, amniotic fluid, sputum, and exhaled breath condensates. Several of the arrays have also been validated for use with ex vivo treated whole blood. EDTA plasma and ex vivo treated whole blood had higher levels of background signal and lower sample pass rates than other matrices.

### Applying Standardized Approaches to Data Redaction

A significant advantage of array-based immunoassays is the ability to measure each analyte in a sample many times. Removal of outlier replicates is obligatory for microarray assays due to signal-related and morphology-based artefacts typically associated with dispersing small volumes of material on a solid substrate in a microarray format. Application of standardized statistical approaches for data redaction is superior to manual inspection and removal of outliers since operator-dependent subjectivity is minimized and throughput is greatly increased. The data redaction procedures described herein employed two, separate steps: Bland-Altman plots and linear correlation analysis. Bland-Altman plots were employed first and identified 99% confidence intervals for all collected data points. This enabled rapid identification and elimination of the outlying 1% of the data with minimal human intervention. This was determined to be an objective, reproducible redaction procedure that greatly reduced time and effort associated with the subsequent, second data redaction step of linear correlation analysis. Linear correlation analysis required performance of 3 replicate assays on each sample, and manual inspection of the series of 3 scatter plots generated from pair-wise correlations of these 3 sample replicates. Individual replicate points for each specific analyte that fell outside the R^2^> 0.95 range were eliminated. In order for data from sample replicates to pass and be admitted into the final data set, the overall sample replicate-to-replicate correlation for the 25–37 analytes of the array was required to have an R^2^> 0.95. Experience in multiplexed immunoassay measurements in samples across more than 30 research projects indicated the R^2 ^value >0.95 to be routinely achievable and associated with high quality replicate data. In each project, the data lost through these two sequential redaction procedures was typically less than 5% of the total original data. An additional quality metric to assess the overall run performance was that at least two of the three replicates must have passed for 85% of the total samples. Runs falling short of this metric were failed and subject to repeat. The typical run fail rate was less than 3%.

### Within-Run Controls to Normalize Data

Within-run controls were employed to account for the effects of systematic variation in replicate measurements. Variation was identified at three levels based on the unique configuration of the 16 sample well microarray chip. The lowest level of variability was observed between the quadruplicate spots of an individual analyte measured within a single sample well. The next level of variation was described as the difference between replicate analyte values measured in different wells located within the same slide. The highest level of variation was associated with measurements taken from a single sample applied to multiple wells positioned across different slides within a run. Since slide-to-slide variation demonstrated the highest system variation, a series of four controls were designed to minimize the impact on sample replicate measurements. The four controls contained all analytes for that array at four concentrations spread across the dynamic range. The four controls were run on every slide within a project and used to generate a global average of total analyte signal. Based on the global average, each individual slide was assigned an adjustment factor to compensate for the slide specific intensity bias. The analyte signal from each individual slide could then be scaled by the adjustment factor to normalize the intensity values between the sample replicates positioned across different slides. In addition, it is possible to use a blocking experimental design, intentionally positioning sample replicates across different slides and different slide locations to eliminate the potential for a slide-specific or location-specific intensity bias. An example of the latter might have been the well at the corner of a slide. Replicate measurements in conjunction with a mechanism to normalize systemic variation results in the production of high quality data required for maximal sensitivity in the identification of significant differences between samples in multiplexed immunoassays. An additional benefit of inclusion of standardized controls run across all slides of every project is the ability to standardize data, for example in mass units, and enable data comparisons between runs, between days and between projects. Such comparisons are necessary when projects constitute large numbers of samples or when it is desired to create a relational database of assay results. Our platform described herein, for example, can perform triplicate measurements on up to 200 samples in a single run.

### Stringent Quality Control of Reagents and Arrays

Quality control of approximately 1200 individual reagents is necessary in order to provide consistent performance of 170 immunoassays on the array platform described herein. These reagents, unfortunately, have widely different shelf life and storage conditions. Stringent quality control procedures specifying performance metrics associated with these reagents were required to achieve reproducible array performance across hundreds of slide lots and reagent sets. Each new lot of a given component was benchmarked to an earlier lot to verify performance. Analyte intensity, dose-response curve, LLD/ULD absolute values, dynamic range and background signal were evaluated in fuctional tests performed on all assay components. Historical performance was monitored by comparing running averages obtained from earlier lots to prevent performance change over time. These procedures were made practical by assembling cocktails of reagents for each step in an assay, dispensing these in single-use aliquots, establishing optimal storage conditions and shelf life, and performing regular (typically weekly) quality checks on aliquots. Implementation of such procedures required use of a laboratory information management system.

### Real-Time Monitors of Platform Performance

The utility of integrating real-time platform performance monitors cannot be understated. Given the complex nature and potential instability of biological reagents associated with a multiplex antibody array, it is critical have a program in place to evaluate performance beyond the quality control release. Real time monitors measure performance of controls under conditions identical to the test samples and reflect a second level verification of assay performance. Our test system employed a series of monitors to capture precision metrics that would create a flag to review the data if the specifications were not met. The requirements included mean coefficients of variation of assay values for controls be less than 15% and for sample replicates be less than 25% for project samples run within a batch. Failure to achieve these metrics indicated a problem related to the performance of the manufactured slides and/or reagents or a technical failure associated with sample handling or assay execution. 15-point standardized titrations were also performed on 6 slides in every run in order to captured detail related to analyte dynamic range, LLQ/ULQ values, dose response behaviour, and background signal that provided a comprehensive assessment of real time platform performance. The detail of the performance assessment was included in final reports for each project to verify data quality and generate confidence in the data generated from a highly complex assay.

### Evaluating the Utility of Multiplexed Immunoassays in Quantitative Proteomics

Evaluating data generated from multiplexed immunoassays for utility in systematic identification of significant differences between samples, or "biomarker discovery", is an important step in understanding the true platform performance. One of the procedures that revealed the sensitivity of the platform for biomarker discovery was variance decomposition analysis for each project. Variance decomposition analysis examines the magnitude of individual components of platform variation and how they compare to analyte variation between samples or individuals. In our experience the platform error of the system described herein was generally an order of magnitude lower than the heterogeneity observed between samples or individuals of the same test group. The utility of this test is in revealing the extent to which platform error impacts the ability to discover moderate expression level differences between samples that are reflective of biological change. Platforms with lower precision will have less sensitivity for detection of relevant differences between samples and will discovery only a subset of the markers that would have been identified with a more precise system.

Finally, a global performance assessment should be performed across multiple projects covering diverse disease areas to gain a solid understanding of the platform utility. An evaluation of this type can be used to identify assays that will not identify differences in expression between samples because they are not sufficiently sensitive, unable to generate sufficient dynamic range given the window of expression, or reveal high endogenous abundance producing assay saturation artefacts. In addition, specific assays that have appropriate sensitivity and dynamic range may be constitutively expressed and therefore poor biomarker candidate analytes for certain disease or treatment effect studies. This analysis may be used to direct efforts to continue to optimize the survey platform in order to generate the highest value in identifying biomarkers using a quantitative proteomic approach.

## Conclusions

Protein microarrays offer the ability to simultaneously survey multiple protein markers in an effort to develop expression profile changes across multiple protein analytes for potential use in diagnosis, prognosis, and measurement of therapeutic efficacy. The current report details certain minimal standards, use of which was found to be necessary to generate the requisite specificity, sensitivity and reproducibility to discover biomarkers. Results revealed that a multiplex system could be operated with high analyte specificity, adequate detection sensitivity and sufficiently broad dynamic range to capture expression differences across diverse disease and therapeutic areas.

## Methods

### Slide Manufacture

#### Glass inspection

Raw soda-lime glass slides (1" × 3") prepared with a Teflon mask configured to provide 16 individual sample wells and an etched barcode for traceability were subjected to visual inspection to identify imperfections that might translate into printing and/or scanning artifacts. Slides with scratches, surface contamination or defects in the applied Teflon mask were identified through a visual examination using a long wavelength inspection lamp equipped with a 532 nm filter. The inspection also failed slides that did not meet stringent dimensional specifications, required for downstream printing and automated assay conditions.

#### Surface activation

Slides passing the visual inspection were silanized with 3-cyanopropyltriethoxysilane according to procedures previously described [17]. Measurements of water contact angle were taken at six discrete locations across the slide surface over a 2% batch sampling to evaluate the uniformity of the applied surface. Since the mean value of contact angle measurements can be influenced by external factors, the deviation in measurements within a batch was also evaluated as an indicator of surface uniformity. Slide batches achieving a mean contact angle value of 52 ± 5 degrees and an average standard deviation of less than 3 degrees were considered suitable for printing.

#### Printing arrays

Capture antibodies prepared as previously described [11] were printed onto coated slides using a PerkinElmer SpotArray Enterprise piezoelectric, non-contact arrayer housed in a class 10,000 controlled access cleanroom. Quadruplicate spots of ~350pL of each capture antibody were applied to each of the 16 wells within a slide generating 256 printed elements per well, 4096 spots per slide and 108 slides per print batch.

### Controls

#### Printed features

Each printed array contained 256 spots representing 64 individual elements printed in quadruplicate. Each array contained 25–40 capture antibodies spread across production chips 1–6, generating a panel of 170 survey analytes. The balance of the elements was reserved for internal assay controls. Each printed array contained multiple copies of an element called BLANK, containing the components used in capture antibody preparation. Blanks were used to survey non-specific sample background within each well. Other printed controls included a series of biotinylated mouse IgG calibration standards to monitor RCA signal amplification and a third control that acted as a monitor for spot contamination resulting from carry-over between sequentially printed features.

#### 15-point standard titration calibrators

Preparations of standardized multiplex analyte titration series were manufactured using recombinant analytes diluted in buffer that covered the range from 12 pg/mL up to 81 ng/mL at 14 discrete points along with two zero analyte buffer blanks. These titration points were distributed among the sixteen available wells on a slide (Figure 1). The standard titrations, designed to overlap the linear range of detection for each individual analyte, were used to generate standard curves from which sample analyte concentrations were determined. The standardized titrations were utilized in both the quality control testing performed on each print lot prior to release, as well as within each client project to verify real-time analyte performance. Six replicates for each point were run in the quality control testing of each slide lot and three replicates of each point were run within each client study to generate standard curves.

#### Anchor point calibrators

The three replicates tested for each study sample were positioned across different slides to avoid slide specific signal bias. Four of the fifteen standard titration points identified as "anchor" points were run across four wells of each sample slide to allow for data normalization of the replicates. The four specific points selected for each array were intended to capture the linear range of detection across the dose response curves for the individual panels of analytes. The remaining 12 wells of the slide were reserved for study samples.

### Slide Qualification

#### Microscopic Examination

Microscopic inspection of printed spots was performed on a 10% sampling of slides within each print batch. Slide selection was biased to interrogate slides located at critical positions on the arrayer deck, reflecting the beginning, middle and end of the print run. Printed slides were examined under a light microscope to evaluate spot positioning, morphology and print grid alignment within each well. Print lots demonstrating features with poor spot morphology, missing spots or misaligned features were not released for use.

#### Positional confirmation

Slides were subject to a full assay function test to confirm the proper location of the printed capture antibodies. Each printed slide lot was tested to confirm the position of the individual feature by spiking in purified analytes in groups of 1–3 per well at a fixed analyte concentration, and performing the full RCA assay to confirm signal at the appropriate printed location. Slide lots revealing signals in inappropriate locations, printing defects or missing signals failed the positional QC test and were not released for use.

#### Performance assessment

Functional testing was performed on a 10% sampling of each slide lot to evaluate the performance of each analyte. Replicates of the 15-point standardized cytokine titration series, were run to evaluate analyte dose-response, average fluorescent signal intensity, replicate spot variability, replicate sample correlations and LLQ/ULQ values to establish functionality of individual features as well as overall array performance for each slide lot produced. Values obtained for the various metrics were compared to historical averages to identify deviations in performance for the individual analytes. Slide lots were failed if analyte dose-response curves produced sample correlations with R^2 ^values less than 0.90, if average replicate spot-to-spot CVs were >15%, or if RCA signal amplification as measured by the biotinylated mouse IgG calibration standards fell below predefined MFI cutoffs.

### Assay

#### Assay Automation

The manual RCA microarray immunoassay reported previously was modified to optimize performance on an automated platform (Protedyne BioCube). Manual immersion washes were substituted with pipette delivered solutions finely tuned to control pipette tip aspiration and delivery position above printed slide wells and to carefully control liquid application and aspiration speeds to minimize disruption to the assembled immunosandwich complex. Incubation times were increased from 30 to 45 minutes for two of the assay steps (RCA signal amplification and detector incubation) and the number and volume of washes between steps increased from 2 to 4–5 and from 20 uL to 30 uL respectively. A Tecan LS200 unit was used to scan the slides. Microarray images were quantified using image capture software (ImageGrabber) developed in-house.

### Clinical Samples

#### Sample procurement/processing

Frozen serum samples from over 800 clinical patients were thawed, centrifuged to remove particulate matter and mixed with 0.25 mg/ml Heteroblock (Omega), 0.25 mg/ml IIR (Bioreclamation) and 0.1% Tween-20 prior to the assay. Twenty microliters of serum was applied to each well.

### Data processing

#### Outlier removal

Data points producing outlier events as a result of missing spots, spots with poor morphology, or printed features demonstrating high pixel outliers were removed using a combination of automated and manual methods. MvA plots, were generated by plotting the difference of the log intensities (M=log_2_(Rep1/Rep2) versus the average of the log intensities (A=log_2_((Rep1*Rep2))/2) for each of the replicates across all analytes. Patterns were visualized using fitted curves from robust local regression with applied visual cues to identify a 99% confidence interval. All outliers in the MvA plot outside of the interval (having a p-value < 0.01) were automatically removed from analysis. The MvA scatter plots also allowed the user to highlight subsets of points on the plot and investigate patterns of intensity differences observed between replicate values. In cases where redaction of an entire replicate (comprised of 4 individual spots) was too stringent, individual spots could be removed using an in-house developed software tool (Terminator) to visually inspect aberrant data points. Data redaction using this method was performed on a limited basis to remove individual spot outliers with poor circularity, non-uniform pixel intensity or missing spots.

#### Sample replicate correlation QC

As a quantitative QC measure, data review included a sample replicate correlation assessment with a predefined correlation coefficient (R^2^) value cutoff. An ideal microarray, when compared to its identical replicate, would have a R^2 ^value of 1. Any comparison producing values lower than the cutoff would result in at least one failed replicate. Individual sample correlations were generated by plotting analyte MFI values (on a Log_2 _scale) from each replicate against the other replicates individually covering all combinations of replicates over the 25–37 analytes within the array. The R^2 ^values obtained for the three plots were manually reviewed to identify failed sample replicates. Only sample replicates with R^2 ^values >0.95 for replicates run within a day or R^2 ^values >0.90 for replicates run across multiple days passed the correlation QC. A summary of the overall sample replicate pass rate monitored the number of failed replicates observed across each of the individual arrays. Project performance specifications required that >85% of all study samples had at least 2 reported replicates.

#### Data Normalization

Individual sample values were normalized using linear regression of the anchor points run across 4 wells of each sample slide to reduce assay imprecision observed among replicates. A four-point standard titration was run on every slide for normalization and quality control purposes. Fluorescence intensities of the four spot replicates for each analyte within a well were averaged on a logarithmic (base two) scale to generate within-slide titration curves. Linear regression coefficients (slope and intercept) were calculated between individual titration curves from each slide to generate an "average" titration curve. Calculated slope and intercept were used to transform averaged analyte values for each sample well. Data normalization was performed on the data set after outlier removal.

#### Precision assessment

A standardized precision assessment was performed on each run to monitor assay performance with respect to; within well variation (based on mean coefficient of variation (CV) observed between quadruplicate printed spots for all features across all sample wells of a project) and between-slide variation (reflecting the average CV observed between all sample replicates across all samples in a study). The mean and median CVs with standard deviations were also metrics included in the precision assessment. The precision assessment was performed as a quality control using the 15-point titration calibrators to qualify new slide lots and generate quality metrics for each client project.

#### LLQ/ULQ determinations

Mean fluorescent intensity (MFI) values, on a logarithmic scale, from the 3 replicate measurements of the 15 point standard titration series were used to generate precision profiles to define the upper and lower limit of quantitation (ULQ, LLQ) within a predefined concentration CV [18]. To do this, a dose-response curve was fitted to the 15-point calibrators using 4-paramter logistic curve fitting procedures. The MFI standard deviation (SD) of the triplicate measurements was converted to concentration SD for the 15 concentration units by dividing by the slope of the dose-response at each concentration point. The conversion provides the relative SD or %CV as a function of analyte concentration to define the precision of the assay for each analyte throughout the working range.

#### Variance decomposition analysis

The VARCOMP procedure of SAS (SAS Institute), was used to obtain estimates of the variance components in a mixed model. The fixed effect variable represents variance observed in different groups in the study, for example groups of healthy versus diseased individuals. Random effects were represented by unique sample identifiers nested within levels of a fixed variable. This component of variance represented within-group differences associated with patient-to-patient variability or disease heterogeneity. The residual variance represented the platform error.

### New Array Validation

#### Establishing analyte sensitivity

Assay sensitivity was determined in two series of experiments. Initial testing to identify analyte cross-reactivity was performed in a configuration where all printed capture antibodies are surveyed in a "1 × n" format, representing a single recombinant analyte tested against all (n) detectors across multiple matrices (serum, heparin plasma, citrate plasma, EDTA plasma and buffer). Capture antibodies that revealed binding to non-cognate antigens were removed or replaced with suitable alternatives. Analytes that demonstrated low signal across all matrices were removed. If signals were low in buffer, a comparison was made with signals obtained in serum or plasma to determine if the endogenous analyte level was detectable and determine if depressed signals were due to analyte instability in a non-biological matrix. Assessment of analyte endogenous level was performed using a "0 × n" format where unadulterated serum and plasma (heparin, citrate, EDTA) are assayed with the full complement of detector antibodies for a given array.

#### Evaluating cross reaction

Two conditions were examined to study potential cross-reactivity between the complex detector antibody mixture and the immobilized capture analyte. The first condition included a 1 × (n-1) format in which 1 analyte was tested in the presence of all detectors minus the detector antibody specific to the added analyte (n-1). In the case of an array containing 40 printed features, 40 unique detector antibody cocktails are prepared containing 39 of the detector antibodies found in the complex mix, with each mix containing all but one of the 40 corresponding detectors. The 40 individual reaction mixes are added to specific arrays after the arrays were incubated with the antigen corresponding to the missing detector. The second condition represented the 0 × (n-1) format where no analyte was added in the presence of all detectors minus the detector specific to the analyte under examination. In each case the analytes were spiked in buffer, serum, and plasma (heparin, citrate, EDTA) at a fixed analyte concentration of 50 ng/ml.

#### Stress testing

Single analyte titrations were prepared in buffer, serum and plasma (heparin, citrate, EDTA) to assign a minimum detection limit (MDL) for each analyte based on a 95% confidence interval above background. The format of the experiments included an n × n design, where all analytes were run in the presence of all detectors. Then, using a 1 × n format, where only one antigen was added to an assay containing all detectors (n), each analyte was tested at 0X, 10X, 50X, and 100X MDL across the same test matrices to identify non-cognate cross-reaction under high analyte load. Additional antigen titration experiments were run to compare the performance achieved in the presence of a single antigen (1 × n) to one in which all analytes were present (n × n).

## List of Abbreviations

MFI: Mean Fluorescence Intensity.

RCA: Rolling Circle Amplification.

CV: Coefficient of Variation

R^2^: correlation coefficient

LLQ: lower limit of quantitation

ULQ: upper limit of quantitation

MDL: minimal detection limits

SD: standard deviation

## Competing interests

L.T. Perlee, J. Christiansen, B. Grimwade, S. Lejnine, V. Tchernev and M. Sorette were employees of Molecular Staging, Inc. and D.D. Patel and S.F. Kingsmore have received consulting fees.

## Authors' contributions

RD oversaw the manufacturing of reagents, JC established standardized quality control testing procedures, MS implemented assay automation and oversaw all clinical testing. BG was responsible for data curation, SL performed statistical analysis. MM and WS contributed to array develoment. SFK, VTT and DDP were involved in study design, LTP in project execution and LTP, SL, JC and SFK in manuscript preparation.
